# Targeted Therapy in the Treatment of Pediatric Acute Lymphoblastic Leukemia—Therapy and Toxicity Mechanisms

**DOI:** 10.3390/ijms22189827

**Published:** 2021-09-11

**Authors:** Monika Lejman, Kinga Kuśmierczuk, Kinga Bednarz, Katarzyna Ostapińska, Joanna Zawitkowska

**Affiliations:** 1 Laboratory of Genetic Diagnostics, Medical University of Lublin, A. Gębali 6, 20-093 Lublin, Poland; lejmanm@poczta.onet.pl; 2 Student Scientific Society, Laboratory of Genetic Diagnostics, Medical University of Lublin, A. Gębali 6, 20-093 Lublin, Poland; kinga.kusmierczuk@gmail.com (K.K.); kbedna.98@gmail.com (K.B.); katarzyna1ostapinska@gmail.com (K.O.); 3 Department of Pediatric Hematology, Oncology and Transplantology, Medical University of Lublin, A. Gębali 6, 20-093 Lublin, Poland

**Keywords:** acute lymphoblastic leukemia, pediatric, molecular toxicity, tyrosine kinase inhibitors, target therapy, immunotherapy, side effects

## Abstract

Targeted therapy has revolutionized the treatment of poor-prognosis pediatric acute lymphoblastic leukemia (ALL) with specific genetic abnormalities. It is still being described as a new landmark therapeutic approach. The main purpose of the use of molecularly targeted drugs and immunotherapy in the treatment of ALL is to improve the treatment outcomes and reduce the doses of conventional chemotherapy, while maintaining the effectiveness of the therapy. Despite promising treatment results, there is limited clinical research on the effect of target cell therapy on the potential toxic events in children and adolescents. The recent development of highly specific molecular methods has led to an improvement in the identification of numerous unique expression profiles of acute lymphoblastic leukemia. The detection of specific genetic mutations determines patients’ risk groups, which allows for patient stratification and for an adjustment of the directed and personalized target therapies that are focused on particular molecular alteration. This review summarizes the knowledge concerning the toxicity of molecular-targeted drugs and immunotherapies applied in childhood ALL.

## 1. Introduction

Acute lymphoblastic leukemia (ALL) is the most commonly diagnosed pediatric neoplasm that belongs to the family of hematologic malignancies. It arises from the uncontrolled proliferation of immature precursor lymphocytic cells originating in the bone marrow. In the United Kingdom between the years 2015 and 2017, a total of 803 ALL cases were diagnosed. A share of 60.3% of all newly diagnosed ALL cases were found in the pediatric population aged 0–19 years, which suggests a strong correlation with patient age. The highest incidence rate per 100,000 people was found among patients aged 0–4, of approximately 5.5 in females and 6.5 in males. On the contrary, among people aged 15–19, the incidence rate per 100,000 people decreases to 1.0 and 2.1 cases in females and males, respectively. Data indicate that ALL is more often diagnosed in the male population than in females [1,2]. Based on immunophenotyping, ALL is classified into two main subgroups, i.e., those derived from the precursors of B or T lymphocytes, both of which involve different subgroups of genetic abnormalities. B-cell ALL accounts for approximately 85% of all childhood leukemias, whereas T-cell ALL occurs rarely and represents 15% [2].

### 1.1. Conventional Therapy

The current high rate of overall survival among pediatric acute lymphoblastic leukemia patients is impressive and exceeds 90% [3,4,5]. A recent report by the Children Oncology Group stated that the overall survival rate for children with standard-risk (SR) B-cell ALL is approximately 96%, whereas event-free survival (EFS) is 89% [4]. Subsequently, the international Associazione Italiana di Ematologia Oncologia Pediatrica-Berlin Frankfurt Munster (AIEOP-BFM) study group reported that OS and EFS for pediatric ALL accounted for approximately 91.9% and 81.4% of cases, respectively [5]. This is an outstanding outcome in comparison with the 1980s, when EFS was 57% [2]. Improvement in overall cure rates can be attributed to the application of treatment protocols based on multi-agent systemic chemotherapy, which includes the usage of vincristine, L-asparaginase, corticosteroids, antimetabolites (cytarabine and methotrexate), and anthracycline [6]. However, conventional therapy is directly linked to a variety of severe side effects, in particular: opportunistic infections, mucositis, neuropathy, bone toxicities, thromboembolism, sinusoidal obstruction syndrome, endocrinopathies, nephrotoxicity, pancreatitis, and hyperlipidemia. The positive impact on the treatment outcome and decreased incidence of drug-related toxicities can be attributed to more precise risk stratification; improvement in supportive care with the use of wide-spectrum antibiotic and antifungal drugs; and, finally, hematopoietic stem cell transplantation (HSCT) in high-risk pediatric ALL groups (HRG-ALL) [7]. Scientists from the Ponte di Legno toxicity working group performed an overall evaluation of acute lymphoblastic leukemia treatment protocols. This particular initiative addressed 14 severe, but relatively rare toxicities, namely: hypersensitivity to asparaginase, hyperlipidemia, osteonecrosis, asparaginase-associated pancreatitis, arterial hypertension, posterior reversible encephalopathy syndrome, seizures, depressed level of consciousness, methotrexate-related stroke-like syndrome, peripheral neuropathy, high-dose methotrexate-related nephrotoxicity, sinusoidal obstructive syndrome, thromboembolism, and Pneumocystis jirovecii pneumonia. The results of the analysis indicated that about half of the ALL pediatric patients undergoing an anti-malignancy regimen will be affected by at least one of the 14 toxicities [8]. Recent reports have suggested that further intensification of first-line chemotherapy does not improve cure rates among high-risk (HR) pediatric ALL patients, but leads to a higher frequency of premature deaths caused by toxicity [7,8]. Therefore, emphasis should be engaged on precise patient treatment stratification based on prognostic features. Currently, prognostic factors predicting the outcome in children with ALL are immunophenotype, response to steroids, genetic alterations, and status of minimal residual disease (MRD).

### 1.2. Minimal Residual Disease

One of the crucial steps in hematological therapy is sequential minimal residual disease (MRD) monitoring. The MRD status is evaluated with the use of sensitive techniques, such as multiparameter flow cytometry (MFC) and polymerase chain reaction (PCR), which enable quantification in more than 90% of ALL pediatric patients [9]. Currently, MRD is the strongest independent prognostic factor of risk stratification in different ALL subtypes, including B- and T-cell lineages, and ALL Ph-positive (Ph+). The measurement of the MRD level can be used to refine the treatment of ALL by identifying patients who require more intensive therapy while sparing others from the risk of unnecessary treatment related toxicities [10]. If the response is delayed or inadequate as assessed by two contemporary techniques for the detection of MRD, patients are considered “early high risk”, and are eligible for treatment escalation or alternative approaches. This depends on the immunophenotyped and on the exact genetic subtype. Regarding the MRD, a large retrospective analysis has been performed. Stanulla M. et al. described a new subgroup of B-ALL, named *IKZF*1^plus^, which is defined as *IKZF*1 deletion co-occurring with deletion in *CDKN2A* or *CDKN2B* (only homozygous deletion), or the *PAX*5 or PAR1 region (*P2RY*8-*CRLF*2), in the absence of *ERG* deletion. The *IKZF*1^plus^ prognostic effect differed noticeably in the patients stratified by MRD levels after induction treatment: 5-year event-free survival for MRD standard-risk *IKZF*1^plus^ patients was 94 ± 5% versus 40 ± 6 10% in MRD intermediate- and 30 ± 6 14% in high-risk *IKZF*1^plus^ patients. The results showed that *IKZF*1^plus^ remains a new MRD-dependent very-poor prognostic factor in risk stratification during ALL therapy [11].

The evaluation for MRD is not only used to evaluate the response to treatment and risk of relapse; after transplantation it is a reliable marker for predicting impending relapses and can thus serve as the basis for pre-emptive therapy in patients after allo-HSCT [12]. The detection MRD is critical for selecting further treatment strategies (immunotherapy and allo-HSCT) for patients with relapsed/refractory B-ALL [13,14]. Recently, new data on the value of MRD post chimeric antigen receptor T cell therapy can help predict the risk of disease relapse, which has therapeutic implications on which patients may benefit from remission consolidation with allo-HSCT [15].

### 1.3. Cytogenetic and Molecular Aberrations with Prognostic Impact

Contemporary advances in whole genome sequencing and single genotype polymorphism genotyping (SNP) provide insights into the molecular background and internal signaling pathways that play a crucial role in the process of leukemogenesis [16,17,18,19]. In 2016, the World Health Organization distinguished a series of recurring genetic alterations that can be detected through conventional standard cytogenetic analysis, fluorescent in situ hybridization (FISH) methods, and microarray or next-generation sequencing (NGS) [20]. The two most common genetic lesions with favorable treatment outcomes are *ETV*6*/RUNX*1 translocation and high hyperdiploidy in B-cell ALL [16]. Due to their excellent prognosis based on MRD status measured during and at the end of the induction phase, patients with *ETV*6*/RUNX*1 translocation or high hyperdiploid ALL can be treated with conventional chemotherapy regimens with a reduced intensity [21,22]. At present, there are no reliable genetic markers that can be used to identify subsets of T-ALL patients that might benefit from reduced-intensity chemotherapy [22,23].

Several genetic abnormalities indicating higher risk failure of treatment have also been identified. ALL Ph+ is a rare subtype in children and accounts for 2–3%, and 25% in adults [16,19,24]. Identification of the *BCR-ABL*1 fusion gene has been associated with a poor prognosis among pediatric patients and, in the pre-tyrosine-kinase inhibitors (TKIs) era, survival was 62% [24]. Incorporation of the therapy of molecular TKIs significantly improved treatment outcomes [25,26]. However, due to the potential resistance mechanisms and long-term molecular toxicity, further clinical trials concerning TKIs are essential. At present, research studies are focused on determining whether adding bispecific monoclonal antibodies, such as blinatumomab, to TKI treatment may result in a better molecular response. Another genetic aberration with a dismal cure rate is *KMT2A*-rearranged ALL, which constitutes a potential target for immunotherapy through the use of blinatumomab and CART-T cell therapy [21,22]. Newly identified provisional entity Ph-like acute lymphoblastic leukemia (Ph-like ALL) accounts for approximately 15% of children with HR B-cell ALL and 10% of SR B-cell ALL subgroups, and is associated with a wide gene expression profile, adverse clinical features, and poor therapy outcome. The incidence of Ph-like ALL increases with age, with a peak in adolescent and young adults, reaching up to 27% cases of young adult B-cell ALL [27]. The results of the study by Pui et al. showed that despite contemporary treatment, patients with hypodiploid ALL continue to have poor overall outcomes with an eight-year survival rate for the entire cohort of only 57.5% [28]. A recent preclinical study identified Bcl-2 as a key therapeutic target and demonstrated the efficacy of a selective Bcl-2 inhibitor, venetoclax, in hypodiploid ALL, providing a promising treatment strategy to improve the outcomes in this subgroup [29].

Recent findings have started an era of new, precise molecular-targeted therapies, providing opportunities to replace conventional chemotherapy with novel antileukemic agents that could decrease chemotherapy-related toxicity. The circumstances listed above may raise some questions; for example, why implement chemotherapy and put patients at risk if there are more suitable and safer methods such as targeted therapy?

This review summarizes the knowledge concerning toxicity during the use of molecular-targeted drugs and immunotherapies applied in childhood ALL.

## 2. Tyrosine Kinase Inhibitors

Protein tyrosine kinases play a crucial role in the regulation of the participation of the cell cycle in cell growth, proliferation, survival, differentiation, and apoptosis processes. Alterations of the tyrosine kinases through chromosomal rearrangements or viral transduction lead to dysregulation and uncontrolled cell signalling [30]. Fusion between the *BCR* and *ABL*1 genes leads to constitutive phosphorylation of GRB2/GAB2, CRKL, JAK/STAT family members, MAPK, and PI3K/AKT pathways [30]. Imatinib is a first-generation ABL1 tyrosine kinase inhibitor with a molecular activity against BCR-ABL1, PDGFR, and c-KIT [31]. The molecular mechanism of imatinib depends on the competitive inhibition of the ATP-active site by binding and changing its conformation, and thus preventing BCR-ABL1 autophosphorylation [30]. Schultz’s studies were crucial for the registration of imatinib as a tyrosine kinase inhibitor in children with Ph+ ALL. The AALL0031 international study of the Children’s Oncology Group (COG) revealed that the five-year disease-free survival for pediatric Ph+ ALL treated with intensive chemotherapy plus imatinib reached 70 ± 6%, and EFS reached approximately 80 ± 11%, compared with the results of the previous Pediatric Oncology Group (POG) treatment protocols without TKI, in which the EFS outcome amounted to around 35 ± 4%. Generally, the authors reported no major toxicities induced by continuous dosing of imatinib at 340 mg/m^2^/d with intensive chemotherapy, although Grade ≥3 toxicity neutropenic infection, fewer total white blood cells, hypokalemia, and hypertransaminasemia were observed [32,33]. A prospective, intergroup, open-label NCT00287105 clinical trial (EsPhALL2010) enrolled a large group of 155 Ph+ ALL pediatric patients between January 2010 and December 2014 for chemotherapy in combination with imatinib, given continuously from day 15 of the induction phase. The study revealed that imatinib-based treatment might increase the incidence of adverse effects. The major forms of toxicity were infections (39%), pancreatitis (6%), and osteonecrosis (5%) [25]. A Polish study conducted between 2012 and 2019 enrolled 31 pediatric patients with newly diagnosed Ph+ ALL treated with an EsPhALL2010 chemotherapy backbone with the addition of imatinib at a dose of 300 mg/m^2^. The authors reported the severity of adverse events of imatinib therapy was mild and mostly Grades 1–2. The most frequently reported forms of toxicity were infections (38.7%), hepatotoxicity (35.5%), and gastrointestinal disorder (22.5%) [26].

The use of imatinib has definitely improved outcomes of the treatment, but the problem of resistance emerged. The mechanism of imatinib resistance is multifactorial and consists of three main processes, namely: mutation of kinase domain BCR-ABL1 fusion, amplification of the BCR-ABL1 oncoprotein, or activation of an additional transduction pathway [34]. To overcome resistance-inducing ABL1 kinase domain mutations, dasatinib and nilotinib, two second-generation tyrosine kinase inhibitors, were developed. Dasatinib is a 325-fold more potent agent than imatinib and, due to its activity against SRC kinase, can play an important role in the treatment of imatinib-resistant mutants. Moreover, dasatinib can cross the blood–brain barrier which is an advantage in targeting leukemia cells in the central nervous system [35]. The safety and efficacy of dasatinib have been studied in the Chinese Children’s Cancer Group’s first randomized, phase III clinical trial that compared clinical outcomes and toxicities between groups of pediatric Ph+ ALL patients treated with imatinib (*n* = 97) and dasatinib (*n* = 92). The comparison showed infections occurred more frequently in the imatinib group in comparison with dasatinib (50% to 38%, respectively). On the contrary, pleural effusion, pancreatitis, and fungal infection appeared more often in the dasatinib group. The authors proved that therapy in the dasatinib group had an excellent treatment outcome relative to that of imatinib, especially in the case of patients with central nervous system infiltration [35]. The DASISION study is an open-label, phase III trial (NCT00481247) with a large group of CML-positive adult patients (*n* = 259). In the five-year follow-up, the study revealed a higher occurrence of treatment-related pleural effusion and pulmonary hypertension in the dasatinib arm. In the imatinib arm (*n* = 260), adverse effects were mainly mild (Grades 1 and 2) with a higher occurrence of edema, muscle spasms, and vomiting. EFS and OS rates remained high and comparable between the two treatment arms; however, in the dasatinib arm, patients achieved treatment milestones faster [36].

Dasatinib displays an affinity to multiple sites within the ABL kinase. Dasatinib has activity against the majority imatinib-resistant BCR-ABL1 point mutations, but has no activity against the T315I mutation [37]; thus, the development of third-generation tyrosine kinase was crucial for this group of patients. Ponatinib is the first third-generation TKI approved by the FDA (in 2014) for treating adults with Ph+ ALL resistant to previous generations of TKIs [38]. Ponatinib demonstrates potency against BCR-ABL1 point mutations, including T315I alteration. Ponatinib’s mechanism of action is characterized by direct inhibition of multiple kinases [39]. A recently conducted single-center, phase II, single-arm trial (NCT01424982) enrolled 76 adult patients with previously untreated ALL Ph+. The patients received eight cycles of ponatinib in combination with hyperfractionated cyclophosphamide, vincristine, doxorubicin, and dexamethasone (hyper-CVAD), alternating with high-dose methotrexate and cytarabine. The data obtained showed that the three-year EFS reached 70%, and the most common reported adverse effects were infections, hepatotoxicity, pancreatitis, and hypertension. The implementation of ponatinib into treatment may also be associated with an increased risk of arterial vascular events [40]. Millot et al. retrospectively collected data from eleven pediatric CML and three ALL Ph+ patients treated with ponatinib in combination with standard chemotherapy as second- to eighth-line treatments. The results showed that the main toxicities in pediatric patients were hematologic toxicity, abdominal pain, headache, and fatigue. No vascular events were reported in this group of patients. Due to the small number of patients, the correlations between ponatinib and the occurrence of side effects were not revealed [41]. The rarity of pediatric Ph+ ALL and the lack of completed clinical trials concerning the usage of tyrosine kinase inhibitors indicates an urgent need for further studies that report on the treatment-related toxicity.

Ph-like ALL represents an ALL subgroup with a B-cell gene expression profile similar to that of ALL Ph+, but without a *BCR-ABL*1 fusion gene. The occurrence of Ph-like ALL increases with age, which in pediatric B-ALL patients amounts to around 12–15%, and is more frequently diagnosed among high-risk ALL subgroups (age > 10 years or white blood cell count ≥ 50,000/uL at diagnosis) defined by the National Cancer Institute (NCI) [27]. The vast majority of Ph-like ALL patients represent a poor outcome, with the five-year EFS amounting to about 41%, and patients mostly harboring a positive MRD status after the end of induction, despite conventional chemotherapy [27]. Ph-like ALL is characterized by a spectrum of genetic alterations activating tyrosine kinase (ABL class fusions and involving other rare kinases: NTKR3, BLNK, DGKH, PTK2B, FLT3, FGFR1, and TYK2), cytokine receptor genes (JAK-STAT signaling pathways), and sequence mutations (FLT3, IL7R, and SH2B3 are the most common) [27,42,43]. Among the other half of pediatric Ph-like ALL subgroups without CRLF2 rearrangement, 15–20% harbored ABL-class gene fusions including ABL1, ABL2, PDGFRA, PDGFRB, LYN, and CSF1R lesions, which are potential targets for an ABL-class first-generation tyrosine kinase inhibitor—imatinib—or a second-generation multikinase ABL1/SRC inhibitor—dasatinib [27,44,45,46]. The frequency of *IKZF*1 deletions was found to be 68% among Ph-like ALL patients of all ages; 81% in adults and 73% in a group of Japanese pediatric patients, in contrast with 15% among non-Ph-like ALL patients [27,39].

In in vitro or patient-derived xenografts, Ph-like cells with ABL-class rearrangements have shown sensitivity to TKIs, such as imatinib and dasatinib. Researchers have reported the clinical data of 24 patients (age range 5–72 years) with Ph-like ALL, who were treated with chemotherapy plus TKIs, both during frontline treatment (*n* = 19) and at relapse (*n* = 5). A total of 14 patients used imatinib—nine had dasatinib and one had ponatinib. The median follow-up was 36 months (range 8–73 months), and 12 patients were alive at the first complete remission, six patients relapsed, and three patients received an alternative TKI, including one in association with inotuzumab (anti-CD22 antibody). TKIs were tolerated well, and only one patient died due to sepsis, which occurred in disease progression [46].

A retrospective, cohort study included 122 pediatric patients with newly diagnosed B-ALL with ABL-class fusion (ABL1, ABL2, CSF1R, and PDGFRB). The results proved that these patients had poor outcomes when treated with regimens that did not contain a tyrosine-kinase inhibitor, despite the use of high-risk chemotherapy regimens and frequent HSCT upon first remission. The five-year EFS was 59.1% (95% CI 50.5–69.1), five-year OS was 76.1% (68.6–84.5), the five-year cumulative incidence of relapse was 31.0% (95% CI 22.4–40.1), and MRD at the end of induction therapy was high in most patients. These outcomes were inferior to those of other pediatric patients who received contemporary treatment protocols. Moreover, the results obtained from this study (*n* = 59) showed that deletions in *IKZF*1 in wild-type ABL-class fusion ALL patients presented more reliable prognostic properties than *IKZF*1 deletions with or without additional deletions in *PAX*5 or *CDKN2A/B.* This Ponte di Legno study proves that the outcome for children with ABL-class fusion is highly unfavorable without TKIs. Den Boer M. et al. established the outcome standard to which these TKI-containing therapies could be compared [47].

More than 50% of pediatric patients with Ph-like ALL harbor CRLF2 (cytokine receptor-like factor 2) rearrangements, in which the majority have coexistent Janus kinase gene mutations, such as JAK1 and JAK2, leading to the activation of the JAK/STAT signaling pathway. Other alterations that are involved in the JAK-STAT pathways described in Ph-like patients are EPOR, JAK3, IL7, SH2B3, and TYK2. Recently conducted in vitro studies have demonstrated that the active JAK-STAT pathway in patient-derived xenografts exhibits a sensitivity to the JAK inhibitor ruxolitinib [48,49,50]. Ruxolitinib is a selective JAK inhibitor with a potential antiproliferative activity. The mechanism of action of ruxolitinib relies on stopping the transduction signal by binding to an active site of the tyrosine kinase JAK1 and JAK2. The FDA authorized the administration of ruxolitinib in 2011 for the treatment of myelofibrosis, and it has subsequently been the focus of treatment of hematological malignancies [51]. Pemmaraju et al. described adverse events of 27 patients older than 14 years, with relapsed or refractory acute myeloid leukemia (*n* = 26) or B-cell acute lymphoid leukemia (*n* = 1), who received ruxolitinib. The most common non-hematologic Grade ≥ 3 toxicity was infection, which occurred in 21 of 27 patients. These infective complications were pneumonia, sepsis, viral infection, and cellulitis. The most common non-hematologic Grade ≥ 3 events were thrombocytopenia (*n* = 10) and neutropenia (*n* = 9) [52]. A non-randomized phase II clinical trial (NCT02723994) of the Children’s Oncology Group (COG) is currently underway, which is investigating the efficiency and safety profile of ruxolitinib in children, adolescents, and young adults aged 1–21 with newly diagnosed B-ALL with CRLF2 and JAK2 pathway alterations [53]. Table 1 presents the toxicity profile in ALL children treated with specific kinase inhibitors.

Due to the excellent response and treatment outcome in Ph+ patients to TKI, there is a strong rationale to conduct further clinical trials on the administration of the combination of standard cytotoxic chemotherapy with specific tyrosine inhibitors based on the genetic kinase mutations present in Ph-like ALL patients [25,32,33,47].

To date, there is a lack of a single optimal treatment protocol for patients with the above-mentioned kinase alterations. The diverse spectrum of genetic landscapes in Ph-like ALL subgroups indicates an urgent need for international cooperative studies to develop standardized treatment protocols with the incorporation of molecular target therapy to improve overall survival.

## 3. Immunotherapy

Novel approaches within the oncology field have revolutionized the course of ALL therapy among adult and pediatric patients. The reason behind the significantly better outcomes in hematological treatment is targeted immunotherapy. The CD19 molecule (expressed in all B-lineage cells) remains the objective of ALL-targeted therapies. Emerging therapies that target the CD19 antigen are based on molecularly modified antibodies and T cells. To date, the FDA has approved four of these; however, certain therapies have predominated the clinical trial market. The abovementioned emerging approaches for childhood ALL are bispecific monoclonal antibodies (BiTEs) and chimeric antigen receptors CART-T cells [56,57].

### 3.1. Blinatumomab

Bispecific CD19-directed CD3 T-cell engagers (BiTEs) are a subclass of bispecific antibodies that recognize their target antigen through dual binding. Blinatumomab was the first registered bispecific monoclonal antibody. Targeting both CD3 and CD19 molecules has proven to be beneficial in ALL patients. Molecularly, BiTEs redirect T cells against tumor cells and block certain mediators of pathologic pathways that contribute to the growth and the persistence of tumors.

The NCT01471782 trial encompassed 49 subjects in phase I and 44 subjects in phase II. It is important to highlight that before the actual drug implementation, subjects underwent an intensive pre-treatment regimen. Surprisingly, two of three Ph+ patients achieved CR. However, primary analysis of the results indicated that of the 70 subjects, only ± 27 (39%) reached complete remission status, of which 14 (20%) were MRD negative. The patients displayed a wide range of toxicities characteristic of immunotherapy. Initially, four dose-limiting toxicities were noted (indicating that four subjects were excluded during cycle 1, phase I). The most common toxicities among the 70 patients were pyrexia (80%), anemia (41%), nausea (33%), and headaches (30%). Seven (10%) of the 70 cases, including three due to allo-HSCT (allogeneic HSCT) after blinatumomab-induced remission, were fatal. The onset of cytokine release syndrome (CRS) (various grades) was registered in eight (11%) patients. Seventeen (29%) patients suffered from neurological-related toxicities and reported adverse effects including tremors, dizziness, somnolence, neuralgia, and higher-grade confusion. B cell deficiency due to blinatumomab implementation was managed via immunoglobulin infusions. Regarding the aforementioned trial, blinatumomab met the requirements to treat pediatric B-cell ALL; however, the CR rate, in addition to the number and degree of toxic events, may indicate the need to improve the BiTE technology and to conduct more trials (and thus, obtain more statistically significant results) [57].

To estimate the effects of blinatumomab, a RIALTO study (NCT02187354) was conducted. Its primary objective was to analyze the incidence of therapy-related adverse effects in children (up to 17 years old) with relapsed/refractory ALL. The full analysis set comprised 110 subjects and almost all of these (99%) experienced adverse events (AEs; of which 74% experienced treatment-related AEs). There were nine fatal AEs (not study-related). A group of patients (42%) experienced neurological AEs, predominantly headaches, with one case of seizures and a depressed level of consciousness. Two patients experienced Grade 3, and one patient experienced Grade 4 CRS. Overall, 58 patients (59%) achieved CR within the first two blinatumomab cycles. Among the CR group, 39 (67%) achieved full hematologic recovery and 46 (79%) achieved MRD response. After evaluation of the results (2019), the safety profile of the RIALTO trial was determined to be safe [58].

Recruitment is currently underway for phase II clinical trials of blinatumomab in combination with sequential chemotherapy (recruiting children and adults with newly diagnosed B-ALL, ID: NCT02877303) and sequential dasatinib (recruiting adults with newly diagnosed Ph+ B-ALL, ID: NCT02744768), as well as one phase III clinical trial of concurrent TKI and blinatumomab, recruiting adults with newly diagnosed B-Ph+ ALL (ID: NCT04530565) [59].

Recently, blinatumomab has reached a number of milestones. It has been proven that a therapy regimen based on blinatumomab results in an event-free survival rate (displayed by a median of 22.4 months of follow-up) among pediatric ALL patients. In comparison with the chemotherapy-regimen group, the bispecific antibody presented a significantly improved SR [60]. Another crucial study resulted in an overall survival of 71.3% for the blinatumomab group vs. 58.4% for the chemotherapy group (data obtained during a time span of two years). Despite impressive results, post-reinduction blinatumomab treatment did not result in a statistically significant difference in disease-free SR. However, the study assessment was limited due to early termination [61]. The main conclusion in both of the aforementioned studies is that the experimental arms with blinatumomab resulted in an overall superior outcome. Blinatumomab-based therapy was also associated with a lower toxicity profile in comparison with standard chemotherapy groups.

### 3.2. CART-T Cell

In childhood clinical practice, only one CART-T therapy has been approved (Tisagenlecleucel, KYMRIAH). The therapeutical CTL019 construct was proven to be highly efficient in clinical trials of childhood ALL patients [62,63]. Therefore, molecularly engineered T cells constitute a prospective type of management in hematological malignancies [64]. However, although CART-Ts are designed according to clinically approved protocols, this therapy does not remain without flaws. The so-called autologous character of the drug may indicate its reliability and safety during treatment. Nonetheless, no definitive cure exists that targets malignancy without causing any side effects. Drug-triggered toxicity can change the route of therapy, including the occurrence of fatal cases [65]. The main reason for therapy-induced toxicity in cancer patients is off-target effects [66,67]. Despite the implementation of innovative therapies in clinical practice, numerous cases have shown adverse reactions. Toxicity may vary in terms of its severity, and, because higher-degree toxicities may be life-threatening, they need to be thoroughly examined and properly managed [68,69].

Despite the significant improvement in treating ALL, problems remain that need to be overcome. The molecular basis of toxicity caused by autologous therapy remains partially undefined. To date, Tisagenlecleucel^(R)^ is the only therapy approved for r/r B-ALL in pediatric patients. Prior to its FDA approval and market registration, the drug was studied in numerous clinical studies. 

ELIANA, Novartis ^®^ (NCT02435849; the pivotal CART-T trial leading to its FDA registration, Basel, Switzerland) was conducted on patients with relapsed or refractory B-cell acute lymphoblastic leukemia. It was a single-arm, multi-center phase II study initially enrolling 92 patients, among which 75 received CTL019 infusion. The study group ranged from 3 to 25 years old, with a median age of 11. The primary aim of ELIANA was to evaluate the safety of this promising approach and to attain an overall 20% CR rate among the study subjects. The overall remission rate was defined by complete remission or complete remission combined with incomplete hematologic recovery. Secondly, further endpoints included complete remissions characterized by undetectable minimal residual disease. The assessment of results in terms of meeting the abovementioned end endpoints was carried out via physical examination, including the duration of remission and the rate of complete survival. The assessment was strongly based on test results obtained within the trial. Sampling undertaken included that of blood, bone marrow, and cerebrospinal fluid (CSF) [70]. Serious adverse effects observed in the ELIANA study were highly specific for CART-T therapies conducted in r/r B-ALL individuals. According to the ELIANA clinical trial fact sheet, the overall remission rates were 82% (*n* = 50), 83% (*n* = 63), and 81% (*n* = 75) at three, six, and three or more (follow-up) months, respectively [62]. The majority of toxicities concerned adult patients. However, any type of obtained results could contribute to CART-T cell development, the enhancement of their anti-tumor action, and a reduction of undesired toxicities. The initial toxicity caused by CART-T infusion is B cell aplasia. This condition is characterized by extremely low B cell counts [71]. The molecular basis of this anomaly lies in the molecular activity of modified T cells. CART-T cells, being programmed against CD19 antigens, automatically target any cell that expresses it. This is an example of the “on-target, off-tumor” activity of CART-T cells. The administration of engineered T cells causes the onset of aplasia manifestations within 1–3 weeks. Because certain molecules (including CD19) are expressed in both cancer and healthy tissues, CART-T does not distinguish them. Therefore, unaffected cells undergo an anti-malignancy regimen. This type of toxicity occurs in every patient infused with CTL019. Clinical reports indicate that immunoglobulin replacement remains routine practice in B cell aplasia management [65]. Another “on-target, off-tumor” toxicity is cytokine release syndrome (CRS). This is the most common toxicity resulting from CART-T therapy. Cytokine release syndrome displays an excessive activation of immune cells (lymphocytes, macrophages, dendritic cells, etc.) resulting in an abundant release of a large numbers of cytokines, and thus leading to elevated levels of IFN-γ, IFN-α, TNF-α, Il-1, IL-5, and IL-6. Adverse effects of CRS vary extensively; hence, this syndrome can manifest as a wide range of symptoms. Mild CRS presents with a manageable flu-like fever, headache, rash, or fatigue. Depending on the level of cellular activation, CRS can develop into moderate or severe life-threatening symptoms. For instance, major manifestations of CRS include elevated body temperature and systemic inflammatory response, and may develop into cardiac or respiratory malfunction, resulting in organ failure. In the results of CRS-affected patients, the levels of IL-6 and CRP are elevated. Management begins with its proper diagnosis and is based on supportive therapy. CRS evasion can be obtained via the proper dosage of infused CART-T cells or the molecular “switch” approach (temporary change of CART-T targeting that enables the monitoring of its cellular activity) [72]. Mild CRS can be managed with antipyretics and intravenous fluids. Registered guidelines suggest the administration of tocilizumab, or corticosteroids in severe cases of CRS. Granulocyte-macrophage colony-stimulating factor (GM-CSF) acts as a crucial factor in mediating CRS. One of the management approaches applied in CRS is the GM-CSF blockade. This is attained via the administration of mAbs (lenzilumab) or GM-CSF gene knockout during CART-T manufacture (to date, this has only been undertaken in a preclinical attempt) [73]. The last (known) CRS mediators are catecholamines. Recent reports suggest that the inhibition of circulating catecholamines leads to a significant reduction in the number of inflammatory cytokines [74]. Summarizing the subject of cytokine-related toxicity, factors that constitute the basis of CRS are inhibited in a molecular manner to acquire relatively safe CART-T constructs.

Immune effector cell-associated neurotoxicity syndrome (ICANS) is the second most common toxicity emerging from CART-T cell therapy. As in the above-mentioned toxicity (CRS), neurotoxicity manifestation ranges from tolerable headaches or aphasia, to high-risk events such as a seizure, cerebral oedema, or coma. Neurotoxicity is associated with a depressed level of consciousness and psychomotor retardation. There are registered cases of death involving cellular therapy-induced neurotoxicity. The pathophysiology of ICANS remains poorly understood, although it is presumably linked to systemic inflammation that includes elevated levels of IL-1, IL-6, and C-reactive protein. Hence, it is speculated that both ICANS and CRS are based on a cytokine storm and its underlying processes, such as excessive endothelial cell activation [75]. In terms of neurotoxicity, another possible factor has been proposed recently: the presence of anti-CD19 brain mural cells (in conjunction with elevated proinflammatory cytokine levels) has been detected in cerebrospinal fluid. Such an occurrence raises serious concerns about the complexity of CART-T-induced side effects [76]. Analogically to CRS management, neurotoxicity can be limited via the incorporation of smaller quantities of engineered CART-Ts, regarding its maximum efficacy at a minimum dose. Unmanaged neurotoxicity among pediatric patients undergoing a CART-T cell regimen results in cognitive difficulties that lead to major mood alterations, including depression. Fortunately, this state is manageable and is usually reduced within the progress of treatment [77]. Sterner et al. providee the first proof of concept that the neutralization of granulocyte-macrophage colony-stimulating factor (GM-CSF) abolishes toxicities (neuroinflammation and CRS) after CART-T cell therapy and may enhance their therapeutic activity. Their experiments suggest that GM-CSF inhibition with lenzilumab enhances CART-T cell proliferation, antitumor activity, and overall survival in vivo. Based on these results, a phase 2 trial of the combination of CART-T cells and lenzilumab is planned [78].

Table 2 contains the most and least common toxicities in immunotherapy approaches.

## 4. Other Approaches

### 4.1. Inotuzumab Ozogamycin

Inotuzumab ozogamycin (InO) is an antibody−drug conjugate indicated for CD22^+^ adult B-cell ALL. Structurally, InO comprises two parts: the humanized antibody part and the calicheamicin part. As the CD22 receptor is a prevalent therapeutic target in ALL treatment (CD22 is present in >90% malignant B-lineage cells), the monoclonal antibody against CD22 antigen can specifically target leukemic B-cells. The antibody part of InO is covalently bound to N-acetyl gamma calicheamicin dimethyl hydrazine (ozogamycin). Ozogamycin is a potent cytotoxic agent that severs the double-stranded DNA structure, resulting in cell cycle arrest and thus apoptosis [79]. Bhojwani D. et al. reported the use of InO in 51 children with relapsed/refractory ALL. In this heavily pre-treated cohort, complete remission was achieved in 67% of patients with overt marrow disease and 70% of patients were negative for MRD. Grade 3 hepatic transaminitis or hyperbilirubinemia were noted in 6 patients (12%) and Grade 3/4 infections were noted in 11 (22%) patients. No patients developed sinusoidal obstruction syndrome (SOS) during InO therapy; however, 11 of the 21 (52%) patients who underwent HSCT following InO developed SOS. Downregulation of the surface CD22 was detected as a possible escape mechanism in three patients who developed a subsequent relapse after InO [80]. Recently, InO demonstrated its functional properties in a phase 1 clinical trial (EUDRA-CT 2016-000227-71 or ITCC-059 study). The subjects of the trial were affected with childhood R/R CD22^+^ acute lymphoblastic leukemia (age range ≥1 to ≤18 years). The CR rate reached 85% within the single course of InO monotherapy and, furthermore, 100% of patients displayed MRD-negativity. Apart from hematologic toxicities, the main life-threatening adverse events were liver-related (veno-occlusive syndrome, also called hepatic sinusoidal obstruction syndrome). Grade 3 or 4 toxicities—hypoxia, anorexia, elevated levels of blood bilirubin, GGT, and AST—were also present [81]. Other reports on InO-based therapy mentioned IFDs (invasive fungal diseases) among paediatric patients [82].

### 4.2. Bortezomib

Bortezomib was the first drug belonging to the class of proteasome inhibitors approved for the treatment of cancer. The exact mechanism of action is unknown; however, proteasome inhibition suppresses the growth of neoplastic tumors. Bortezomib acts indirectly on the activity of NF-κB, an anti-apoptotic transcription factors whose activity is increased in leukemia cells exposed to chemotherapeutic agents. IκBα, which is a negative regulator of NF-κB, is stabilized by bortezomib, which translates into a reduction of NF-κB transcription, and thus inactivation of its downstream targets. The inhibition of NF-κB transcription makes leukemic cells prone to apoptosis [83]. Bortezomib does not show a clinical activity against ALL on its own, but it is effective in combination with other chemotherapeutic agents used in the treatment. In vitro studies with T cell ALL and B cell ALL have both shown that bortezomib increases the sensitivity of cancer cells to the cytotoxic effects of chemotherapeutic agents [84]. Bortezomib can lead to serious adverse events such as neurological and pulmonary toxicity. Pulmonary adverse events associated with bortezomib include pneumonia, interstitial pneumonia, and acute respiratory distress syndrome [85]. Regarding neurological toxicity events, peripheral neuropathy is most likely to develop after treatment with bortezomib [86]. A group of 37 patients aged 21 years or less was tested with doxorubicin, vincristine, dexamethasone, and pegylated asparaginase, in which bortezomib was administered intravenously at a dose of 1.3 mg/m^2^ on days 1, 4, 8, and 11 of therapy. The most common side effect was microbial infection, occurring between days 15 and 22 after starting treatment. The study reported three patient deaths due to multiple organ failure, each associated with a fungal infection. Sepsis due to *Klebsiella pneumoniae* infection was also reported on day 13. Within the subsequent week, another two patients developed sepsis caused by *Candida parapsilosis* and *Stenotrophomonas maltophilia*. All cases of sepsis were treated with appropriate therapies. Another common side effect was events related to the nervous system. One patient developed central ataxia accompanied by headache, dysarthria, and confusion. After a few days, the symptoms resolved spontaneously. Five patients developed sensory and motor neuropathy. In all of these cases, the symptoms resolved after supplementation with vitamins B1 and B6, and gabapentin. Metabolic disorders such as hypocalcemia, hyponatremia, and hypokalemia were reported in 15 out of the 37 (40.5%) patients. In 83.7% of respondents, decreased levels of fibrinogen and coagulation disorders were demonstrated. During treatment, 35% of the subjects (13 patients) developed bone marrow aplasia. Common symptoms, such as diarrhea, vomiting, and nausea, also appeared among patients, but were controlled with appropriate medications [87]. In a further study, bortezomib was tested in combination with intrathecal methotrexate, mitoxantrone, dexamethasone, vincristine, and pegasparase. As in the study described above, bortezomib was administered intravenously on days 1, 4, 8, and 11 of therapy at a dose of 1.3 mg/m^2^. Ten children with the first or subsequent relapse of ALL participated in the study. Four patients (40%) developed a Grade 3 or higher infection during therapy. A patient who refused further treatment died of a fungal infection. Two patients experienced respiratory failure following infection. Severe pulmonary complications occurred in 20% of patients, but in this case, bortezomib was excluded as a contributing factor. There was no evidence of neurological toxicity of Grade 2 or greater in this study [88]. Another study in Japan of six children tested the effects of bortezomib in two groups treated with different chemotherapeutic drugs. In the first group, bortezomib was combined with vincristine, doxorubicin, L-asparaginine, and dexamethasone (Regimen A). The second group consisted of patients who received bortezomib with vincristine, dexamethasone, mitoxantrone, and L-asparaginase (Regimen B). A patient who received Regimen B in combination with bortezomib died 32 days after initiation of therapy due to respiratory failure, despite intensive supportive therapies. From day 11, the patient developed sensory and motor neuropathy, and was also in a state of neutropenia (<500/µL). The remaining patients developed neutropenia (<500/µL), lasting an average of 22 to 35 days, and thrombocytopenia (<50,000 µL), lasting an average of 30 to 65 days. Most of the patients experienced peripheral neuropathy and neuropathic pain. One developed prolonged peripheral neuropathy and required an additional two years of rehabilitation because the symptoms had not been resolved via bortezomib administration. Additionally, patient 5, who was treated with regimen A and bortezomib, developed bacteremia caused by *E. coli* and *Klebsiella pneumoniae* infection after 14 days of treatment initiation. The same patient developed another bacteremia caused by *Enterococcus feacium* and *Klebsielle pneumoniae* on day 25 after the start of treatment [89]. Bortezomib has been shown to be generally well-tolerated and effective in patients with ALL. However, its side effects suggest that more research is needed to accurately determine any adverse effects that may occur after use. In addition, studies are needed to demonstrate the toxicity of bortezomib in monotherapy, and to thus define its toxicity without the effects of other chemotherapeutic agents with which research has already been conducted.

### 4.3. BCL2 Inhibitors

Proteins from the BCL-2 family are the most important regulators of the intrinsic pathway of mitochondrial apoptosis. They are antiapoptotic and their overexpression indicates a poor prognosis and resistance to treatment. In the treatment of neoplasms associated with proteins from the BCL-2 family or BCL-2 expression, BCL2 inhibitors such as venetoclax or navitoclax are used. Venetoclax is a highly selective BCL-2 inhibitor that is orally bioavailable and is highly potent. It activates caspases, releases apoptotic proteins, and also inhibits the survival targets of cancer cells, which translates into the promotion of apoptosis. Navitoclax is an orally bioavailable inhibitor of BCL-XL and BCL2, and works in the same way as venetoclax [90,91]. In preclinical ALL models, the anti-tumor activity of BCL-2 inhibitors, including venetoclax and navitoclax, was shown [29,92,93,94]. Unfortunately, thrombocytopenia due to BCL-XL inhibition by navitoclax limits the dose at which it can be used [91,95]. Therefore, the focus was on venetoclax, which spares platelets by targeting BCL-2 only. However, studies have been performed in which navitoclax was used in combination with venetoclax.

A phase I study was conducted that investigated the effectiveness and safety of venetoclax alone and in combination with chemotherapeutic agents (decitabine, azacitidine, and cytarabine). The study was conducted on a group of 29 patients aged 1 to 25 years old with AML (*n* = 18) and ALL (*n* = 11). The most common side effects in patients treated with venetoclax in combination with chemotherapy were diarrhea (52%), vomiting (52%), hypokalemia (48%), febrile neutropenia (45%), and anemia (41%). Additionally, increased levels of alanine aminotransferase (ALT) were reported in 48% of patients and increased levels of aspartate aminotransferase (AST) were reported in 45% of patients. Adverse events of Grade 3 or higher in children and adolescents treated with venetoclax monotherapy occurred in 65% of the subjects, and 24% revealed serious adverse events associated with venetoclax. The conclusions obtained in this study indicate a generally good tolerability of the use of venetoclax in combination with chemotherapy; however, its use in monotherapy led to a few cases of dose reduction or even discontinuation of therapy due to the occurrence of side effects [96].

**Figure 1:**
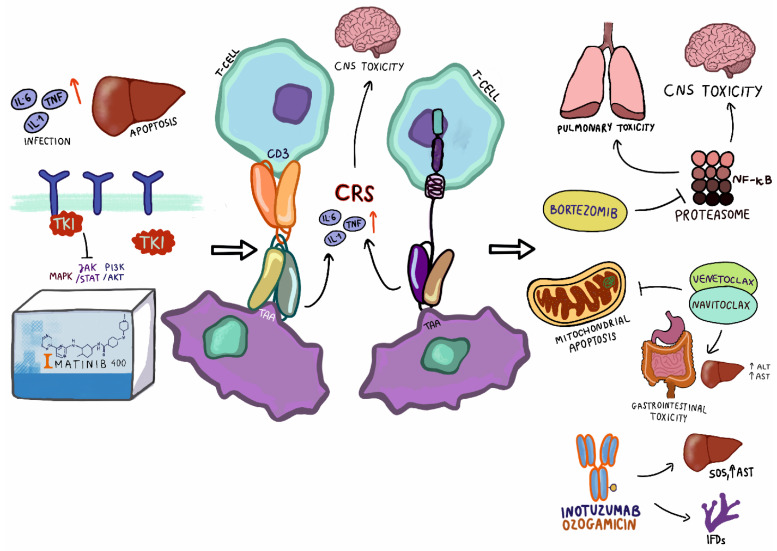
Toxicities associated with targeted therapy in pediatric patients with ALL. TKI—tyrosine kinase inhibitor; TNF—tumor necrosis factor; TAA—tumor-associated antigen; CNS—central nervous system; ALT—alanine transaminase; AST—aspartate transaminase, SOS—sinusoidal obstruction syndrome.

A study on the efficacy and safety of venetoclax in combination with navitoclax was conducted in 29 pediatric and adult patients with lymphoblastic lymphoma (LLy) and acute lymphoblastic leukemia (NCT03181126). The study included 16 patients with T-ALL, 18 patients with B-ALL, and 2 patients with LLy. Among the patients, the most frequent adverse events were nausea (47%), hypokalemia (42%), diarrhea (42%), and vomiting (42%). Additionally, abdominal pain (36%) and febrile neutropenia (33%), in addition to neutropenia and ALT increase, were observed in 31% of patients. Fatal adverse events were reported in three patients, regardless of disease progression. One of these patients experienced cardiac arrest (the patient had previously been diagnosed arrhythmia) and two died of sepsis. Navitoclax-related adverse events that limited the possible dose to be taken included drug-induced hepatitis, intestinal ischemia, and delayed recovery. There were no significant pharmacokinetic interactions between navitoclax and venetoclax in the study [97]. As a relatively new therapeutic method in the treatment of ALL, the use of BCL2 inhibitors requires further studies on the effectiveness and toxicity of these inhibitors in therapy. At this time, two clinical trials are planned in which venetoclax will be tested. The first is a study on idasanutilin in combination with venetoclax, to investigate its pharmacokinetics, tolerability, safety, and effectiveness in children and young adults (NCT04029688). The second study will investigate the safety and pharmacokinetics of venetoclax in pediatric patients with relapsed or refractory malignances (NCT03236857). Figure 1 presented toxicities associated with targeted therapy in pediatric patients with ALL.

## 5. Conclusions

This article provides a summary of the available literature concerning the toxicity of targeted therapies applied in ALL pediatric patients. The combination of chemotherapy and tyrosine kinase inhibitors in children with ALL significantly improves the prognosis and survival rates. Most patients treated with TKIs do not experience serious adverse events. The most commonly reported infections, gastro-toxicities, and cytopenias are manageable with supportive therapy. JAK/STAT inhibitors are new, potentially effective drugs that can be administered to treat Ph-like B cell ALL. An assessment of the safety profile of these inhibitors is still under investigation in pediatric patients. Novel adoptive immunotherapy (CART-T cells and BiTE antibodies) targeted at the patient’s own immune components displays a wide range of side toxicities. In addition to the expense of CART-T-based therapy, the main obstacles are toxicities based on excessive cytokine release (cytokine release syndrome and neurotoxicity), which can be managed via ad hoc approaches. BiTE therapy demonstrates a similar profile of cytokine-dependent side effects. CART-T, in addition to BiTE approaches, requires further research and modification to be maintained as a secure option for B-ALL pediatric patients. Toxicities concerning bortezomib include metabolic disorders, in addition to neurological and pulmonological toxicities. Among BCL2 inhibitors, the majority of toxic events lie within the febrile neutropenia, hypokalemia, or gastric dysfunction. Inotuzumab Ozogamycin is a potential future milestone of second line treatment in children with BCP-ALL in association with Blinatumomab. Available data on targeted therapies (including clinical trials) enable estimation of the toxicities induced by ALL novel therapies among pediatric patients. However, to obtain more significant results and to gain knowledge on toxicity mechanisms, further testing is needed.

## Figures and Tables

**Table 1:** Comparison of most common toxicities of specific kinase inhibitors associated with targeted therapy in patients with ALL.

Drug	Molecular Target	Common Toxicities	Uncommon Toxicities	References
Imatinib	BCR-ABL1	Infections, mostly bacterial	Osteonecrosis	
	c-KIT	Gastrointestinal toxicity	Joint pain Rash/skin problems	[25,26,31,32,33]
	PDGFR	Superficial edema Nausea Muscle cramps Musculoskeletal pain	Headache	
Dasatinib	BCR-ABL1c-KIT PDGFR SRC family FGFR1 EGFR	Pleural effusions Myelosuppression	Bleeding (GI ^1^, PR ^2^) Hemorrhage	[31,35]
Nilotinib	BCR-ABL1	AEs ^3^ related to bilirubin increases Headache Pyrexia	Nasopharyngitis Fatigue Abdominal pain Nausea Vomiting	[35,54,55]
	c-KIT			[6,51]
	PDGFR			
Ponatinib	BCR-ABL1 T315I	Platelet toxicities Nausea Abdominal pain	Infection Leucopenia Neutropenia	[45]
Ruxolitinib	JAK1 JAK2	Infection (pneumonia, sepsis) Thrombocytopenia Neutropenia	Stroke Cerebral edema Fatigue Mucositis	[52,53]

^1^ GI, gastrointestinal; ^2^ PR, rectal; ^3^ AEs, adverse events.

**Table 2:** Toxicity profile of CART-T and blinatumomab therapies.

Immunotherapy	Molecular Target	Common Toxicities	Uncommon Toxicities	References
Chimeric Antigen Receptor (CART-T) T cells	CD19	B cell aplasia Cytokine release syndrome Infections Immune effector cell-associated neurotoxicity syndrome	Febrile neutropenia Tumor lysis syndrome	[72,75,76]
Blinatumomab (BiTE antibody)	CD3 and CD19	Anemia Nausea Neurotoxicities (headache, tremor, dizziness, and higher-grade confusion)	Cytokine release syndrome	[57,58]
